# Closing Remarks for the Special Issue “Local Healthcare Preparedness and Alert Systems—How to Prevent Future Pandemics?”

**DOI:** 10.3390/epidemiologia7050136

**Published:** 2026-09-28

**Authors:** Giuseppe Stirparo, Alberto Arnedo-Pena

**Affiliations:** 1 SIMED—Società Italiana di Medicina e Divulgazione Scientifica, Via Trento 41, 43121 Parma, Italy; 2 PrimeCare Centro Medico Leonardo, Via Locatelli 2, 24050 Bariano, Italy; 3 Public Health Center, 12003 Castelló de la Plana, Spain; albertoarnedopena@gmail.com

The publication of this Special Issue marks the conclusion of a collection dedicated to the topic of local healthcare preparedness and alert systems, with the aim of exploring strategies capable of strengthening health systems and preventing or mitigating the impact of future pandemics. This topic is considered the top-ranked research priority for epidemic preparedness, including AI-based outbreak prediction, improved diagnostics for infectious diseases, and early-warning systems. In the context of global health [1], infectious diseases pose potential threats of epidemics and pandemics [2,3].

The contributions gathered in this issue highlight the importance of integrating epidemiological surveillance, healthcare organization, policy frameworks, and innovative care models to improve resilience at the local level. A total of 24 articles were submitted, highlighting the importance of this topic for our scientific community and for readers of the journal Epidemiologia.

A total of 14 articles were published in this Special Issue, offering multidisciplinary perspectives on infectious disease preparedness and response. From a geographical standpoint, most contributions originated from Southern Europe: eight studies (57%) were conducted in Italy, three (21%) in Spain, two (14%) in the United States, and one (8%) in Brazil, reflecting both strong regional engagement and international collaboration. This factor is particularly understandable because Italy was the first Western country with a high spread of COVID-19 and the first country that took restrictive measures to control the pandemic situation [4], which is why it had to quickly develop various models of organizational adaptation. Furthermore, this pandemic has been very significant because COVID-19 has also had a long-term impact on the health of citizens and healthcare systems [5,6,7].

The studies included in this collection were conducted mainly in the recent post-pandemic period (2020–2024), although some contributions analyzed historical or long-term data. In particular, one article explored the plague epidemic in Spain between 1742 and 1743, highlighting the relationship between socio-economic disparities and mortality during infectious disease outbreaks. This historical perspective provides a valuable reminder that social determinants have long played a critical role in shaping the outcomes of epidemics.

A significant proportion of the contributions addressed the COVID-19 pandemic, which continues to represent a key reference point for evaluating healthcare preparedness [5]. Seven articles (50%) focused specifically on COVID-19, examining topics such as the impact of the pandemic on healthcare systems, epidemiological surveillance, risk profiles associated with infection and co-infections, socio-demographic determinants influencing preventive behaviors, and the quantification of excess mortality during the pandemic in Spain.

The methodological approaches were varied, although observational designs predominated. Retrospective observational studies represented the largest group (six articles; 43%), followed by prospective observational studies (two articles; 14%), while other contributions included legislative analyses, conceptual frameworks, a mixed-methods cross-sectional study, and another cross-sectional study.

Four contributions focused on vaccination and prevention strategies, identifying key actions and frameworks to improve adherence to immunization programs and prevent the spread of communicable diseases. These studies highlight the importance of coordinated public health interventions and effective communication strategies to support vaccination uptake.

One study showed that telemedicine could manage patients presenting with respiratory symptoms, suggesting its potential to reduce pressure on healthcare facilities during infectious disease outbreaks. Furthermore, only one contribution evaluated the use of CBRNe personal protective equipment in pre-hospital settings, demonstrating that such equipment does not significantly reduce the operational autonomy of healthcare professionals for the majority of medical procedures.

Finally, two studies examined public health legislation and organizational models, including the definition of alert phases and surveillance indicators for respiratory infectious diseases such as influenza and COVID-19, as well as community-based care models aimed at improving the management of frail elderly populations.

Taken together, the articles in this Special Issue highlight that preparedness for future pandemics requires an integrated and multi-level approach, combining epidemiological evidence, strong governance, technological innovation, and community-based healthcare systems, we resume all the information in Table 1.

As Guest Editors, we would like to sincerely thank all the authors who contributed to this Special Issue, as well as the reviewers for their valuable and constructive feedback. We also thank the editorial team for their support throughout the publication process. We hope that the research presented here will contribute to the ongoing discussion on how to strengthen local healthcare preparedness and alert systems and support more resilient responses to future public health emergencies.

## Figures and Tables

**Table 1:** Main findings in the Special Issue.

Country	Year	Study Design	Main Results	Pathogen
United States [8]	2022	Retrospective observational study	Risk profiles for the development of co-infections	COVID-19, MPXV
Italy [9]	—	Logical Framework	Methods for preventing communicable diseases	Vaccinations
Italy [10]	2022–2023	Prospective observational study	The equipment does not significantly reduce the autonomy of professionals in the pre-hospital setting	CBRNe personal protective equipment
Italy [11]	2022	Narrative Logical Framework	Main actions to increase vaccination adherence	Vaccinations
Italy [12]	2022	Prospective observational study	Through telemedicine, it is possible to manage about 80% of patients with respiratory-infectious symptoms	Patients with respiratory disease
Spain [13]	1742–1743	Retrospective study	Link between socio-economic disparity and mortality	Plague epidemic
Italy [14]	2023–2024	Legislative analysis	Definition of alert phases and indicators for respiratory influenza-like diseases	Influenza–COVID-19–Vaccinations
Italy [15]	—	Legislative analysis	Organizational model of community health houses	Vaccinations-Frail elderly
Spain [16]	2020	Cross-sectional study	Epidemiological Surveillance	COVID-19
Italy [17]	2019–2020	Retrospective observational study	Impact of COVID on the national healthcare system	COVID-19
Italy [18]	2019–2020	Retrospective observational study	Impact of COVID on the national healthcare system	COVID-19
United States [19]	2020	Cross-sectional mixed-methods study	Socio-demographic factors influencing the propensity to adopt behaviors aimed at reducing infectious diseases	COVID-19
Spain [20]	2020–2025	Retrospective observational study	Quantification of excess mortality during the COVID-19 pandemic in Spain	COVID-19
Brazil [21]	2020–2024	Retrospective observational study	The study assessed COVID-19 case fatality and identified the timing of its reduction over the course of the pandemic	COVID-19
